# Effect of Isoenergetic Substitution of Cheese with Other Dairy Products on Blood Lipid Markers in the Fasted and Postprandial State: An Updated and Extended Systematic Review and Meta-Analysis of Randomized Controlled Trials in Adults

**DOI:** 10.1016/j.advnut.2023.09.003

**Published:** 2023-09-17

**Authors:** Rebecca Pradeilles, Tom Norris, Laury Sellem, Oonagh Markey

**Affiliations:** 1 School of Sport, Exercise and Health Sciences, Loughborough University, Loughborough, United Kingdom; 2 Montpellier Interdisciplinary Centre on Sustainable Agri-Food Systems (UMR MoISA), University of Montpellier, CIRAD, CIHEAM-IAMM, INRAE, Institut Agro, IRD, Montpellier, France; 3 Institute of Sport, Exercise and Health, Division of Surgery and Interventional Science, Faculty of Medical Sciences, University College London, London, United Kingdom; 4 Carenity (ELSE CARE), Paris, France

**Keywords:** adults, butter, cardiovascular disease prevention, cheese, dairy structure, fasting lipid profile, dairy matrix, lipids, lipoproteins, saturated fat, whole dairy

## Abstract

Consumption of fat as part of a cheese matrix may differentially affect blood lipid responses when compared with other dairy foods. This systematic review was conducted to compare the impact of consuming equal amounts of fat from cheese and other dairy products on blood lipid markers in the fasted and postprandial state. Searches of PubMed (Medline), Cochrane Central and Embase databases were conducted up to mid-June 2022. Eligible human randomized controlled trials (RCTs) investigated the effect of isoenergetic substitution of hard or semi-hard cheese with other dairy products on blood lipid markers. Risk of bias (RoB) was assessed using the Cochrane RoB 2.0 tool. Random-effects meta-analyses assessed the effect of ≥2 similar dietary replacements on the same blood lipid marker. Of 1491 identified citations, 10 articles were included (RoB: all some concerns). Pooled analyses of 7 RCTs showed a reduction in fasting total cholesterol, LDL-C and HDL-C concentrations after ≥14 d mean daily intake of 135 g cheese (weighted mean difference [WMD]: −0.24 mmol/L; 95% confidence interval (CI): −0.34, −0.15; *I*^2^ = 59.8%, WMD: −0.19 mmol/L; 95% CI: −0.27, −0.12; *I*^2^ = 42.8%, and WMD: −0.04 mmol/L; 95% CI: −0.08, −0.00; *I*^2^ = 58.6%, respectively) relative to ∼52 g/d butter. We found no evidence of a benefit from replacing cheese for ≥14 d with milk on fasting blood lipid markers (*n* = 2). Limited postprandial RCTs, described in narrative syntheses, suggested that cheese-rich meals may induce differential fed-state lipid responses compared with some other dairy matrix structures, but not butter (*n* ≤ 2). In conclusion, these findings indicate that dairy fat consumed in the form of cheese has a differential effect on blood lipid responses relative to some other dairy food structures. However, owing to considerable heterogeneity and limited studies, further confirmation from RCTs is warranted.

**Trial Registration Number:**

This systematic review protocol was registered at https://www.crd.york.ac.uk/PROSPERO/ as CRD42022299748.

**Statement of Significance**

This updated and extended systematic review and meta-analysis investigated whether hard and semi-hard cheese intake differentially affects fasted and postprandial state blood lipid and lipoprotein outcomes among disease-free adults when compared with other dairy matrix structures (including butter and milk). Our main findings indicate that, relative to butter, dairy fat eaten in the form of cheese reduced fasting circulating total cholesterol, LDL-cholesterol, and to a lesser extent HDL-cholesterol and reinforce the notion that the blood lipid responses to dairy-derived SFA differ based on their food matrix structure.

## Introduction

Cardiovascular diseases (CVDs) are the main cause of mortality worldwide and are expected to be responsible for >23.6 million deaths by 2030 [1]. Dietary guidelines traditionally recommended limiting consumption of dietary SFA, as high saturated fat intake has been associated with an increase in LDL-C concentrations, an established risk factor for atherosclerotic CVD [[2], [3], [4]].

Dairy products are a leading contributor to dietary SFA intake in societies with high dairy intake, predominantly Western populations [5]. Dairy generally refers to milk and milk-derived foods, including butter, buttermilk, cheese, cream, and sour cream [5]. It is, however, noted that most dietary national guidelines recommend nutrient-dense products within the “dairy” food group (primarily milk, cheese, and yogurt), and commonly, the low-fat or fat-free varieties of these to limit SFA consumption, with butter often excluded from this food group [[5], [6], [7]]. The traditional approach to evaluating the nutritional value of foods is to classify foods based on the content of a single nutrient (for example, SFA) [8]. However, this reductionist approach fails to consider other nutrients and bioactive components contained within the whole food structure (that is, the food matrix), and how they may interact to alter the overall nutritional and health effects of a specific food [8,9]. More recently, some food-based dietary guidelines have placed emphasis on dietary patterns, with the Dietary Guidelines for Americans [7] considering nutrient adequacy within caloric limits (in addition to SFA content). Nonetheless, food-based dietary guidelines may not fully capture the nutritional and health benefits of whole dairy foods, which are determined by complex interactions between the sum of constituents within the dairy food matrix structure, and their subsequent impact on nutrient bioavailability, absorption, and physiological responses [[10], [11], [12]].

Conventional dairy foods (that is, cheese, milk, and butter) are a heterogenous food group in terms of their fat, protein, micronutrient (for example, calcium [Ca] and phosphorus), milk fat globule membrane (MFGM), bioactive peptide, bacterial starter culture content and physical structure [6,9,13]. The MFGM is a tri-layered membrane rich in bioactive polar lipids (phospholipids and sphingolipids) and proteins enclosing fat globules in unprocessed milk, which is disrupted by food processing techniques and differentially preserved in dairy products [10,14]. Emerging human studies suggest that the MFGM fraction (or membrane-associated milk polar lipids) may have a beneficial impact on the fasting blood lipid profile (including LDL-C concentrations) (for review, see [9,14]). Solid cheese, a fermented (cultured) dairy food, has the most complex dairy matrix, with the fat present in milk fat globules within a solid matrix rich in Ca, milk proteins (mostly casein), and MFGM [9,10,15]. Despite its high-fat content and structural differences, cheese has a composition more comparable to milk and yogurt because of mineral, protein and MFGM contents [9,10]. Conversely, conventional butter (considered in the randomized controlled trials [RCTs] presented in the current review), a water-in-oil emulsion with very low protein, micronutrient, and MFGM contents [9,10,15], is a non-fermented food item. However, butter may come in different types, have a different composition, and can undergo a fermentation process (for example, with the addition of bacterial cultures) [9,16]. The complexity of the dairy food matrix may help to explain why observational studies have shown that greater consumption of full-fat dairy products, except butter, is not adversely associated with CVD risk [9,17,18].

A systematic review and meta-analysis (SRMA) of ≤5 RCTs published in 2015 indicated that isoenergetic replacement of butter with hard or semi-hard cheese significantly lowered fasting circulating total cholesterol (TC), LDL-C, and HDL-C, without affecting triacylglycerol (TG) concentrations [13]. To help inform proposed food-based dietary recommendations [6], there is a need to provide an up-to-date SRMA of well-controlled RCTs that also considers the impact of the cheese matrix on additional lipid biomarkers, including fasting apolipoprotein (apo) B, and postprandial TG concentrations, as well as subgroup analysis according to baseline CVD risk status. Circulating LDL-C is only partly representative of the atherogenic lipid burden and apoB (the total number of atherogenic lipoprotein particles) has been shown to be a more potent predictor of CVD risk than LDL-C concentrations [19,20]. Furthermore, the non-fasted state is important in the context of the pathogenesis and progression of cardiometabolic diseases, with elevated postprandial TG (which may reflect the atherogenic capacity of TG-rich lipoproteins) recognized as an independent CVD risk factor [[21], [22], [23]]. Thus, the current systematic review with meta-analysis addressed the question of whether hard and semi-hard cheese consumption differentially affect fasted and postprandial state blood lipid and lipoprotein concentrations in disease-free adult population when compared with isoenergetic quantities of other dairy foods.

## Methods

This review followed the PRISMA guidelines [24]. A protocol for this SRMA was published in the PROSPERO database (www.crd.york.ac.uk/PROSPERO); Registration number: CRD42022299748.

## Study eligibility criteria

Studies considered for inclusion in this review were full-text, peer-reviewed reports of RCTs published in the English language. The search strategy was not restricted to any publication period. Study eligibility criteria were established by the population or participant, intervention, comparator, outcome, and study design framework (Table 1). RCTs among weight-stable adults aged ≥18 y with no presence of chronic medical conditions (including CVD, type 2 diabetes, or liver diseases) and not taking prescription medication for cholesterol, lipid, or blood pressure lowering were considered for inclusion. RCTs were also required to meet the following criteria to be deemed eligible for inclusion: *1*) the intervention consisted of hard or semi-hard cheese; *2*) the comparator consisted of an isoenergetic alternative dairy food; *3*) presented ≥1 fasting blood lipid or lipoprotein outcome in the fasted or postprandial state. Studies were excluded if >1 dairy food was included in a dietary treatment, if the dairy foods were fortified, and if studies matched for quantity of dairy foods rather than energy content. Exclusion criteria included studies that *1*) only compared cheese consumption to non-dairy food comparators and *2*) used dairy products as a vehicle of fortification (for example, vitamin D, phytosterols, conjugated linoleic acid, and omega-3 fatty acids [FAs]). To be deemed eligible for inclusion, postprandial studies were required to include *1*) a moderate to high-fat meal challenge containing 30–80 g of total fat (with fat-matched intervention and comparator treatment arms) and *2*) assess relevant blood lipid or lipoprotein outcomes over a 4–8-h post-meal observation period to capture the postprandial lipemic response, including the peak TG concentration [[25], [26], [27]].

**Table 1:** Inclusion and exclusion criteria for the review on the impact of isoenergetic substitution of cheese with other dairy products on blood lipid markers in the fasted and postprandial state

Parameter	Inclusion	Exclusion
Population or participants	Human studies including weight-stable adults aged ≥18 y who are apparently healthy or at risk of cardiometabolic disease development (for example, elevated lipid profile, BMI ≥25 kg/m^2^). There were no restrictions regarding sex, ethnicity, or study setting	Studies conducted in adults with chronic diseases for example, cardiovascular disease, diabetes, or liver disease; prescribed medication for cholesterol, lipid, or blood pressure-lowering; non-weight-stable populations (for example, on a weight loss program or >5 kg weight gain or loss in last 6 mo) Studies conducted in animals or pregnant women, children, and adolescents <18 y and in vitro research
Independent variable (intervention)	Dietary intervention with hard- or semi-hard cheese postprandial studies only: the test meal must contain 30–80 g of total fat. High-fat test meals (intervention and comparator) will be required to contain similar amounts of total fat. These studies will also be required to have assessed relevant outcomes over a 4–8-h postprandial period	Studies without non-fortified cheese or including >1 dairy food in the dietary treatment
Comparator	Energy-matched quantity of an alternative dairy food including, butter, milk, and soft or fat-reduced cheese	Studies that only compare cheese consumption to non-dairy food comparators Studies which have used fortified dairy product comparators
Dependent variable (outcome)	Blood lipids and lipoproteins assessed in the *1*) fasted and *2*) postprandial state. Eligible studies will have included ≥1 fasting blood lipid or lipoprotein outcome in the fasted or postprandial state, including circulating apolipoprotein A-I, apolipoprotein B, HDL cholesterol, lipoprotein(a), LDL cholesterol, total cholesterol, triacylglycerol, or VLDL cholesterol, as primary or secondary outcomes	
Study design	Randomized controlled trials	Cross-sectional studies; trials without a control group; case-control studies; pre-post studies without a control; narrative reviews; systematic reviews; and meta-analyses
Publication status	Full-text articles published in peer-reviewed journals	Conference abstracts, conference proceedings, unpublished data, reports, letters, and editorials
Language	English	Articles not published in English

## Search strategy

Literature searches were conducted in 3 major electronic databases: PubMed (Medline), Cochrane Central, and Embase up to mid-June 2022. The search strategy was developed by the review team (OM and RP) and checked by an independent academic librarian. Scoping searches were conducted to refine the search strategy to ensure that relevant studies had been identified with the search syntax. As outlined in our study protocol (CRD42022299748), we originally searched PubMed (Medline), Scopus, and Web of Science databases. However, after discussion with an information specialist and piloting our search strategy, we modified our search strategy in June 2022 to include more relevant databases. The search strategies developed for each database are presented in Supplemental Table 1. The reference lists of review papers identified in our search and included RCTs were also checked for additional publications.

## Study selection

Duplicate records were removed in Covidence software (Veritas Health Innovation) before screening. A multiple-pass method was employed to review the articles identified in the database searches. Selection of studies for inclusion via title and abstract (first pass), and subsequently full text (second pass) was conducted independently by 2 independent reviewers (RP and OM). Any disagreements were resolved by discussion. Reasons for exclusions at full-text screening were recorded.

## Data extraction process

The data extraction form was developed in Excel and piloted by 2 reviewers on 10% of the included fasting and postprandial studies to assess suitability. After the first pilot, the data extraction form was revised and finalized. Data extraction was undertaken by a single reviewer (OM). An independent reviewer (RP) checked a subset of 25% of articles for completeness, accuracy, and consistency, with discrepancies resolved through discussion. Full details of the information extracted from eligible studies are presented in Supplemental Table 2. In brief, extracted data included first author name, publication year, country, study design, study population and participant characteristics, details of intervention and comparator conditions, relevant outcomes (including assessment method, analytical sample size, reporting of power calculation, any adjustment for confounding and measures of intervention effect), and funding source. Means, SDs, SEs, or 95% confidence intervals (CIs) of the change from baseline values, baseline values and post-intervention values were extracted, where possible. For RCTs involving multiple arms, only data from the intervention and relevant comparator were extracted. Study protocols and supplementary materials were searched for data extraction if the required information was not presented in the included articles.

## Risk of bias assessment

Two reviewers (RP and OM) independently assessed risk of bias (RoB) of included articles using the Cochrane RoB tool 2.0 at the results (outcome) level for crossover or parallel RCTs, where appropriate [28]. In brief, each of the 5 domains of bias: *1*) randomization process, *2*) deviations from the intended interventions, *3*) missing outcome data, *4*) measurement of the outcomes, and *5*) selection of the reported results in the tool were rated as being at low, moderate (some concerns), or high RoB, with the inclusion of notes to justify the judgment [28]. For crossover RCTs, the potential RoB arising from period and carryover effects was also assessed. Washout periods of ≥14 d were judged acceptable regardless of the intervention duration [29]. In line with Sellem et al. [29], shorter washout periods were deemed suitable only when combined with interventions of >28 d to ensure ≥14 d of exposure to the dietary treatment with minimal risks of carryover effects. Full-text articles were used as the main source of information for the assessments. Reviewers also checked clinical trial registrations (for example, clinicaltrials.gov), supplemental files, and secondary publications for required information where possible. Subsequently, the 2 reviewers then compared independent ratings for each included RCT, discussed inconsistencies, and reached consensus on each domain. After completing consensus on the domains in the tool, the 2 reviewers agreed on the overall RoB for each study was assessed. The overall RoB score was judged as “low risk” if all domains were rated as low risk, “some concerns” if 1 domain was rated as concerning, but no domain was rated as high risk, and “high risk” if ≥1 domain was rated as high risk or if several domains were scored as concerning in a way that may substantially affect the confidence in the reported results. The overall RoB in each study was summarized using the criteria by Sterne et al. [28] (for details, see Supplemental Table 3). RoB figures for individual studies and a summary of RoB figures were prepared using risk-Of-Bias VISualization tool [30].

## Data synthesis methods

### Eligibility and preparation for synthesis

Outcome data values at the end of the intervention were presented as means ± SDs in SI units. Missing data (timepoint or not presented as mean ± SD) were obtained either by contacting the authors of the original full-text articles [31,32], by converting SEs to SDs [33], or by closest approximation of mean and SD from median and interquartile range values, using previous guidance [34,35]. As no response was obtained by contacting the authors of 1 of the original full-text articles [31], it was necessary to extract change score data from the 2015 SRMA by de Goede et al. [13] (which they retrieved through contact with the original authors). Outcomes reported in the current review were continuous. Thus, intervention effects were measured as weighted mean difference (WMD) between 2 dietary interventions. In line with Cochrane Handbook recommendations [33], we accounted for within-participant variance in crossover RCTs, effect measures, and their SDs in crossover RCTs using correlation coefficients from a crossover trial with 2 4-wk isocaloric dietary intervention periods with palmitic and stearic acid [36], as employed in previous meta-analyses [29]. With the exception of the LDL-C:HDL-C ratio, there was a correlation coefficient value available to correct the effect measures of all reported outcomes. Forest plots were generated for each suitable outcome. If there were insufficient comparable RCTs to meta-analyze effect estimates, a narrative synthesis of quantitative data was conducted in accordance with the SWiM guidelines [37].

### Statistical analyses

A priori, we set a minimum requirement of ≥2 studies reporting the same outcomes to perform a meta-analysis, in line with Cochrane Handbook guidance [33]. Studies were pooled using an inverse variance random-effects model to account for possible heterogeneity. The restricted maximum likelihood (REML) method was employed to estimate heterogeneity. As outlined previously [29], this method is recommended for meta-analyses of continuous outcomes containing <10 studies [38]. In addition, the Hartung–Knapp–Sidik–Jonkman (HKSJ) correction was applied to estimate the 95% CIs of the summary effects [39,40]; this is a more conservative approach for pooling a small number of studies when compared with the Wald-type method [38,41,42]. Statistical heterogeneity was quantified using the *I*^2^ values. We considered an *I^^2^^* *≥*50% as evidence of substantial heterogeneity [33]. In addition, for the cheese compared with butter comparison, subgroup analyses were performed to explore the effects of health status (that is, cohorts classified as “at risk” of CVD according to overweight/obesity status or presence of elevated LDL-C concentrations compared with apparently healthy cohorts) on TC, LDL-C, HDL-C, and TG concentrations. Because of insufficient data, no subgroup analyses were performed on the following outcomes: apoB apoA-I, the TC:HDL-C ratio, and LDL-C:HDL-C ratio.

## Results

### Study selection and characteristics

**Figure 1:**
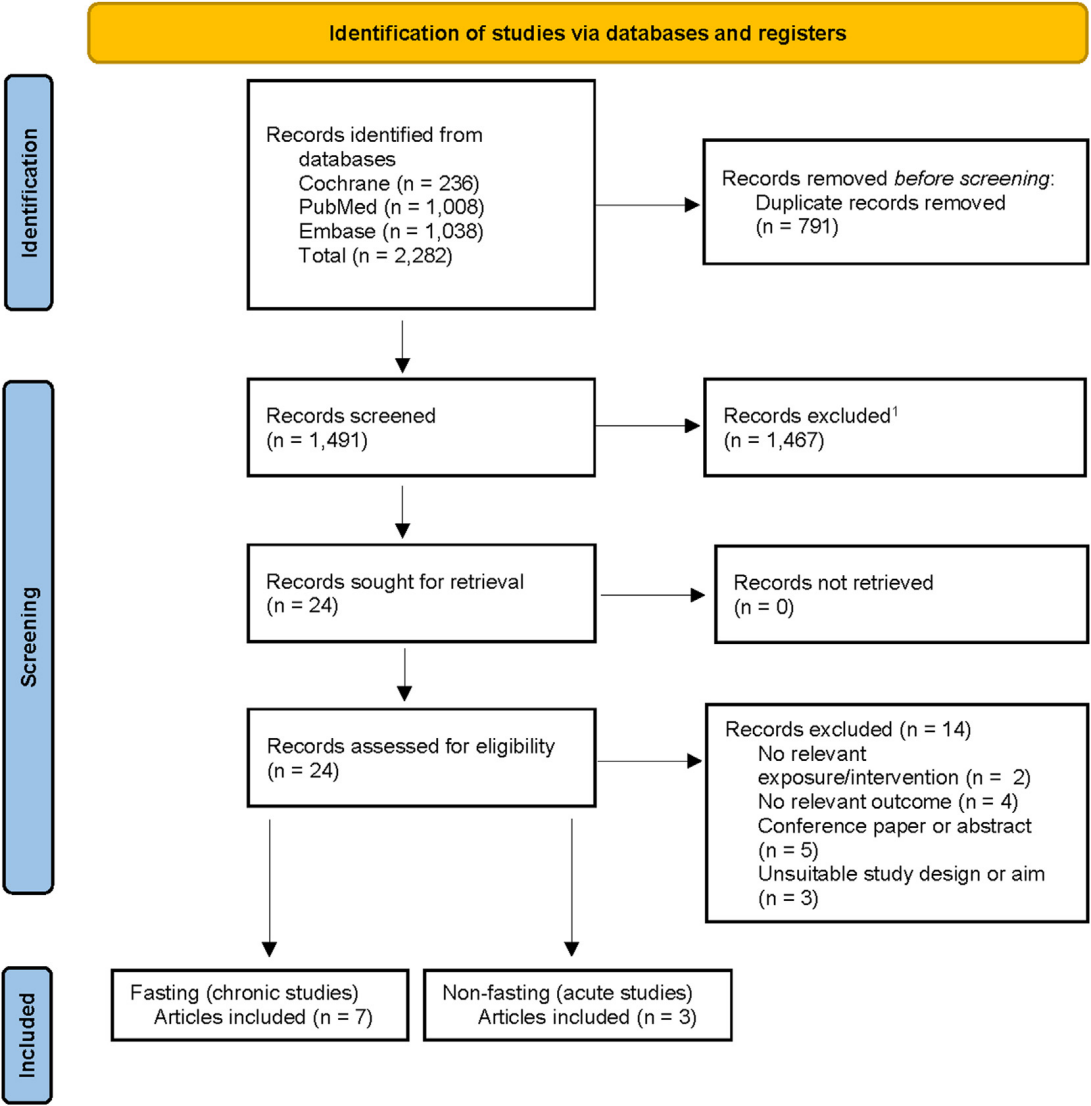
Flowchart of study search and selection for the review of the effect of isoenergetic substitution of cheese with other dairy products on blood lipid markers in the fasted and postprandial state.

The selection process and included RCTs are summarized in Figure 1. We identified 2282 records, of which 1491 were screened after removal of duplicates. After the exclusion of 1467 records at the first stage of screening (title and abstract), 24 records were assessed in detail at the full-text screening stage. A further 14 records were excluded for not meeting the pre-defined inclusion criteria, primarily because of reporting no relevant outcomes (*n* = 4) or based on being a conference paper or abstract (*n* = 5). Overall, 10 full-text articles met the inclusion criteria and were included in the review. The number of full-text articles is lower than in an earlier SRMA by de Goede et al. [13], as we did not compare cheese intake to isocaloric quantities of non-dairy foods, including tofu and other protein-rich foods.

The characteristics and results of included RCTs are presented in Table 2, Table 3 and Supplemental Table 4. Among the 10 included studies, 7 fasting (chronic) RCTs were included in quantitative meta-analyses and 3 fed/postprandial (acute) RCTs were included in the narrative synthesis. One fasting study comparing regular fat compared with fat-reduced cheese appeared to meet the inclusion criteria but was excluded as the study arms were not matched for energy content [43]. Furthermore, it was not possible to compare the regular cheese to fat-reduced cheese intervention arms in Feeney et al. [44], as the latter arm incorporated butter so that the diet periods were matched for energy and nutrient content.

**Table 2:** Synthesis of characteristics of eligible RCTs on the impact of isoenergetic replacement of cheese with other dairy products on blood lipid markers in the fasted state^1^

First author, year (country)	Health status	Study completers, *n* (%, M/F)	Age (mean or range)	BMI (mean or range)	Study design, type of dietary intervention, duration of intervention and washout (d)	Study arms, *n*	Intervention	Comparator	Relevant outcomes	Industrial funding (yes/no)	Overall RoB
Cheese vs. butter											
Tholstrup, 2004 (Denmark) [45]	Healthy	14 (100/0)	23 y	22 kg/m^2^	Crossover, fully controlled, 21-d intervention, ≥28-d washout (habitual diet)	3	205 g/10 MJ Samsø (hard) cheese (45% fat/dry weight): 26% fat and 1989 mg Ca/10 MJ Milk fat contributed to 20%TE (SFA: 16%TE)	64 g/10 MJ butter: 54 g fat + 10 mg Ca/10 MJ Milk fat contributed to 20%TE (SFA: 16%TE)	ApoA-I, apoB, HDL-C, HDL_2_-C, HDL_3_-C, LDL-C, LDL-C:HDL-C ratio, TC, TG, and VLDL-C PO: NR	Yes	Some concerns
Biong, 2004 (Norway) [46]	Healthy	22 (41/59)	23–54 y	19–37 kg/m^2^	Crossover, fully controlled, 21-d intervention, 7-d washout (habitual diet)	3	150 g/d Jarlsberg (hard) cheese (equivalent to 20%TE from total fat; SFA: 12.1%TE); Ca: 2108 mg/8 MJ	52 g/d butter with Ca-caseinate (equivalent to 20%TE from total fat; SFA: 12.1%TE) Ca: 1143 mg/8 MJ	ApoA-I, apoB HDL-C, Lp(a), LDL-C, LDL-C:HDL-C ratio, TC, TG PO: NR	Yes	Some concerns
Nestel, 2005 (Australia) [47]	Elevated plasma LDL-C: >3.6–<6.0 mmol/L	19 (74/26)	56.3 y	27.7 kg/m^2^	Crossover, semicontrolled, 28-d, 14-d washout/run-in (low-fat dairy, moderately increased carbohydrate diet)	2	120 g/d mature Cheddar (hard) cheese (33% fat of wet weight); 40 g/d dairy fat (SFA: NR); Ca: NR; Diet: Total fat: 36.2%TE; SFA: 16.7%TE	∼50 g/d butter; 40 g/d dairy fat (SFA: NR); Ca: NR; Diet: Total fat: 37.4%TE; SFA: 17.2%TE	HDL-C, LDL-C (PO), TC, TG	Yes	Some concerns
Hjerpsted, 2011 (Denmark) [31]	Healthy	49 (57/43)	55.5 y	25.3 kg/m^2^	Crossover, semicontrolled, 42-d, 14-d washout/run-in (habitual diet)	2	143 g/d Samsø (hard) cheese (27 g fat/100 g) at medium energy level. Fat content of cheese replaced 13%TE from dietary fat (SFA: 63.9% by weight) and 34 mg Ca/100g	47 g/d salted butter at medium energy level. Fat content of cheese replaced 13%TE from dietary fat (SFA: 64.9% by weight) and 19 mg Ca/100g	HDL-C, LDL-C, TC, TG PO: NR	Yes	Some concerns
Soerensen, 2014 (Denmark) [48]	Healthy	15 (100/0)	27.2 y	23.1 kg/m^2^	Crossover, fully controlled, 14-d-, ≥ 14-d washout (habitual diet)	3	120 g/d Klovborg (semi-hard) cheese (per 10 MJ); 45% fat/dry weight (SFA: 47.1 g); Dairy Ca: 810 mg/10 MJ; Non-dairy Ca: 362 mg/10 MJ	Butter (quantity NR^2^) (per 10 MJ); (SFA: 45.1 g); Dairy Ca: 0 mg/10 MJ; Non-dairy Ca: 362 mg/10 MJ	HDL-C, LDL-C, TC, TG PO: total excretion of fecal fat	Yes	Some concerns
Brassard, 2017 (Canada) [32]	Abdominally obese: WC: ≥94 and ≥80 cm for M and F, respectively, and low serum HDL-C: ≤1.34 and ≤1.53 mmol/L for M and F, respectively	92 (47/53) - completed ≥1 diet (*n* = 77 post-intervention)	18–65 y	30.6 kg/m^2^ (mean BMI for cheese and butter groups)	Crossover, fully controlled, 28-d, ≥24-d washout (median: 33 d; habitual diet)	5	90 g/2500 kcal full-fat Cheddar (hard) cheese; Diet: Total fat: 32.0%TE; SFA: ∼12.6%TE (mainly from cheese); Ca: 1261.0 mg/2500 kcal	49 g/2500 kcal butter; Diet: Total fat: 32.0%TE; SFA: ∼12.4%TE (mainly from butter); Ca: 811.1 mg/2500 kcal	apoB, HDL-C (PO), LDL-C, TC, TC:HDL-C ratio, TG	Yes	Some concerns
Feeney, 2018 (Ireland) [44]	Overweight	Per-protocol analysis: 127 (NR): *n* = 40 and *n* = 28 for cheese and butter treatments, respectively	50–70 y	≥25 kg/m^2^	Parallel, semicontrolled, 42-d, NA	4	120 g/d full-fat Cheddar (hard) cheese (489 ± 19 kcal/d); Total fat: 40.8 g (SFA: 25.2 g); Ca: 828 mg	49 g/d butter + Ca-caseinate powder (30 g) + 500 mg Ca supplement (CaCO_3)_ (489 ± 19 kcal/d); Total fat: 39.2 g (SFA: 25.9 g); Ca: 817 mg	HDL-C, LDL-C (PO), NEFA, TC, TG	No	Some concerns
Cheese vs. milk											
Tholstrup, 2004 (Denmark) [45]	Healthy	14 (100/0)	23 y	22 kg/m^2^	Crossover, fully controlled, 21-d intervention, ≥28-d washout (habitual diet)	3	205 g/10 MJ Samsø (hard) cheese (45% fat/dry weight): 26% fat and 1989 mg Ca/10 MJ Milk fat contributed to 20%TE (SFA: 16%TE)	1500 mL/10 MJ whole fat milk: 54 g of fat + 1779 mg Ca/10 MJ Milk fat contributed to 20%TE (SFA: 16%TE)	apoA-I, apoB, HDL-C, LDL-C, LDL-C:HDL-C ratio, TC, TG, VLDL-C PO: NR	Yes	Some concerns
Soerensen, 2014 (Denmark) [48]	Healthy	15 (100/0)	27.2 y	23.1 kg/m^2^	Crossover, fully controlled, 14-d, ≥14-d washout (habitual diet)	3	120 g/d Klovborg (semi-hard) cheese (per 10 MJ); 45% fat/dry weight (SFA: 47.1 g) Dairy Ca: 810 mg/10 MJ; Non-dairy Ca: 362 mg/10 MJ	∼670 mL semi-skimmed milk (per 10 MJ; 1.5% fat) (SFA: 46.5 g); Dairy Ca: 781 mg/10 MJ; Non-dairy Ca: 362 mg/10 MJ	HDL-C, LDL-C, TC, TG PO: total excretion of fecal fat	Yes	Some concerns

Abbreviations: apo, apolipoprotein; Ca, calcium; F, females; HDL_2_-C, high-density lipoprotein 2 cholesterol; HDL_3_-C, high-density lipoprotein 3 cholesterol; LDL-C, LDL cholesterol; Lp(a), lipoprotein (a); M, males; NEFA, nonesterified fatty acid; PO, primary outcome; NA, not applicable; NR, not reported; TC, total cholesterol; TG, triacylglycerol; WC, waist circumference; %TE, percent total energy.

1 Fully controlled intervention: all foods consumed were provided to participants, for either home or on-site consumption (on campus, metabolic ward, etc.). Semicontrolled intervention: experimental foods were provided to participants together with dietary advice for nonexperimental foods.

2 Mean daily intake estimated as 52 g/d.

**Table 3:** Synthesis of results of eligible RCTs on the impact of isoenergetic replacement of cheese with other dairy products on blood lipid markers in the postprandial state

First author, year (country)	Health status	Study completers, *n* (%, M/F)	Age (mean or range)	BMI (mean or range)	Study design, duration of washout (d), duration of postprandial period (h)	Dietary intervention arms (n), standardized meal components	Intervention	Comparator	Relevant outcomes	Result	Industrial funding (yes/no)	Overall RoB
Cheese vs. butter												
Drouin-Chartier 2017 (Canada) [50]	Healthy	43 (44/56)	36.9 y	24.7 kg/m^2^	Crossover, ≥2-wk washout, 8 h	3 Bread, icing (topping), fruit juice	Young Cheddar (hard) cheese (32% milk fat): 41.5 g total fat and 33.1 g dairy fat per 1000 kcal serving^1^	Salted butter: 41.5 g total fat and 33.2 g dairy fat per 1000 kcal serving^1^	TG (PO: triglyceride response at 4 h), NEFA, ApoB-48	↔ TG, NEFA or ApoB-48% change in concentrations from baseline to 4 h or percentage difference in iAUC_0–8 h_ between cheese and butter meals	Yes	Some concerns
Hansson 2019 (Norway) [49]	Healthy and OW/OB	47(30/70)	25–46 y	23.6 (21.0–25.8) kg/m^2^	Crossover, ≥2–5 wk washout period, 6 h	4 White bread, raspberry jam	Gräddost (Semi-hard cheese): Full-fat milk- and cream-based cheese: 45.1 g dairy (total) fat per 715 kcal meal	Butter: 44.8 g dairy (total) fat per 629 kcal meal	TG (PO: iAUC_0-6h_), TC, LDL-C, HDL-C, NEFA	↔ TG, NEFA, TC or LDL-C iAUC_0–6 h_ between cheese and butter meals	No	Some concerns
Cheese vs. cream cheese or homogenized cheese												
Drouin-Chartier 2017 (Canada) [50]	Healthy	43 (44/56)	36.9 y	24.7 kg/m^2^	Crossover, ≥2-wk washout, 8h	3 Bread, icing (topping), fruit juice	Young Cheddar (hard) cheese (32% milk fat): 41.5 g total fat and 33.1 g dairy fat per 1000 kcal serving^1^	Cream cheese (31% milkfat, 55% moisture, unripened, homogenized, fresh cheese): 41.5 g total fat and 33.2 g dairy fat per 1000 kcal serving^1^	TG (PO: triglyceride response at 4 h), NEFA, ApoB-48	↔ TG or NEFA% change in concentrations from baseline to 4 h or % difference in iAUC_0–8 h_; ↑ 2 h TG concentration after cream cheese vs. Cheddar cheese (*P* = 0.0004); ↓ 6 h TG response after cream cheese vs. Cheddar cheese (*P* = 0.0004); ↓ 2 h NEFA concentration after cream cheese vs. Cheddar cheese (*P* = 0.04); ↓ apoB-48 iAUC_0–8 h_ (*P* = 0.01) and ↓ apoB-48 response at 4 (*P* = 0.02) and 6 h (*P* = 0.0002) after cream cheese vs. Cheddar cheese	Yes	Some concerns
Kjølbæk 2021 (Denmark) [15]	Healthy	21 (100/0)	19–40 y	20.0–24.5 kg/m^2^	Crossover, ≥2-wk washout period, 8 h	4 Bread and water (for similar density)	Matured (8–12 wk) full-fat (50+) Cheddar (hard) cheese: 66.6 g dairy fat (intertwined network of protein and areas of coalesced fat) and 68 g total fat per standardized 4.7 kJ meal	Homogenized Cheddar cheese: 66.6 g dairy fat (loss of protein network and small discrete fat droplets) and 68 g total fat per standardized 4.7 kJ meal	TG and apoB-48 (PO: iAUC_0-8h_), TC, HDL-C, LDL-C, NEFA, apoB-100	↔ iAUC_0-8 h_ or 8 h concentrations for TG, ApoB-48, TC, HDL-C, LDL-C, NEFA or apoB-100 between cheese meals	Yes	Some concerns
Cheese vs. whipped cream												
Hansson 2019 (Norway) [49]	Healthy and OW/OB	47(30/70)	25–46 y	23.6 (21.0–25.8) kg/m^2^	Crossover, 2–5 wk washout period, 6 h	4 White bread, raspberry jam	Gräddost (Semi-hard cheese): Full-fat milk- and cream-based cheese: 45.1 g dairy (total) fat per 715 kcal meal	Whipped cream: 45.1 g dairy (total) fat per 652 kcal meal	TG (PO: iAUC_0-6h_), TC, LDL-C, HDL-C, NEFA	↔ iAUC_0-8 h_ for TG, NEFA, TC, HDL-C or LDL-C between cheese and whipped cream meals	No	Some concerns
Cheese vs. sour cream												
Hansson 2019 (Norway) [49]	Healthy and OW/OB	47(30/70)	25–46 y	23.6 (21.0–25.8) kg/m^2^	Crossover, 2–5 wk washout period, 6 h	4 White bread, raspberry jam	Gräddost (Semi-hard cheese): Full-fat milk- and cream-based cheese: 45.1 g dairy (total) fat per 715 kcal meal	Sour cream: 45.2 g dairy (total) fat per 655 kcal meal	TG (PO: iAUC_0-6h_), TC, LDL-C, HDL-C, NEFA	↑ iAUC_0-6 h_ for TG and HDL-C after sour cream vs. cheese meal (*P* = 0.02 and *P* = 0.01, respectively) ↔ iAUC_0-6 h_ for NEFA, TC or LDL-C between cheese and sour cream meals	No	Some concerns

Abbreviations: apo, apolipoprotein; NEFA, nonesterified fatty acid; OW/OB, overweight/obese; PO, primary outcome; TC, total cholesterol; TG, triacylglycerol;%TE, percent total energy.

^1^The serving size was adjusted according to individual energy requirements.

#### Fasting studies

Apart from 1 parallel-designed RCT [44], all other 6 fasting RCTs were conducted in a crossover manner [31,32,[45], [46], [47], [48]]. Seven studies compared the substitution of cheese with butter [31,32,[44], [45], [46], [47], [48]], with 2 of these studies also designed to assess the replacement of cheese with milk [45,48]. Six studies intervened with hard cheese (*n* = 2 Samsø; *n* = 1 Jarlsberg; and *n* = 3 Cheddar) [31,32,[44], [45], [46], [47]] and one study used semi-hard cheese (Klovborg) as the treatment [48]. The intervention duration ranged from 14 to 42 d. Most of the RCTs included males and females, except for 2 studies that enrolled males only [45,48]. Four RCTs reported including apparently healthy adults only [31,45,46,48] but 3 RCTs targeted recruitment to specifically include individuals classified as overweight [44], those with elevated LDL-C concentrations [47], or abdominal obesity and low serum HDL-C [32].

#### Postprandial studies

All 3 postprandial studies were conducted in a crossover manner [15,49,50]. One study each compared substitution of hard [50] or semi-hard cheese [49] with butter, and 1 study compared hard cheese with cream cheese [50], homogenized cheese [15], and cream or sour cream [49]. Two studies intervened with hard cheese (*n* = 2 Cheddar) [15,50] and 1 with a semi-hard cheese (*n* = 1 Gräddost) [49]. The blood sampling duration ranged from 6 [49] to 8 h [15,50]. Two RCTs included males and females [49,50], whereas Kjølbæk et al. [15] enrolled males only. Two RCTs included apparently healthy adults only [15,50], whereas Hansson et al. [49] reported recruiting normal weight and individuals classified as overweight/obese.

### RoB **assessment**

**Figure 2:**
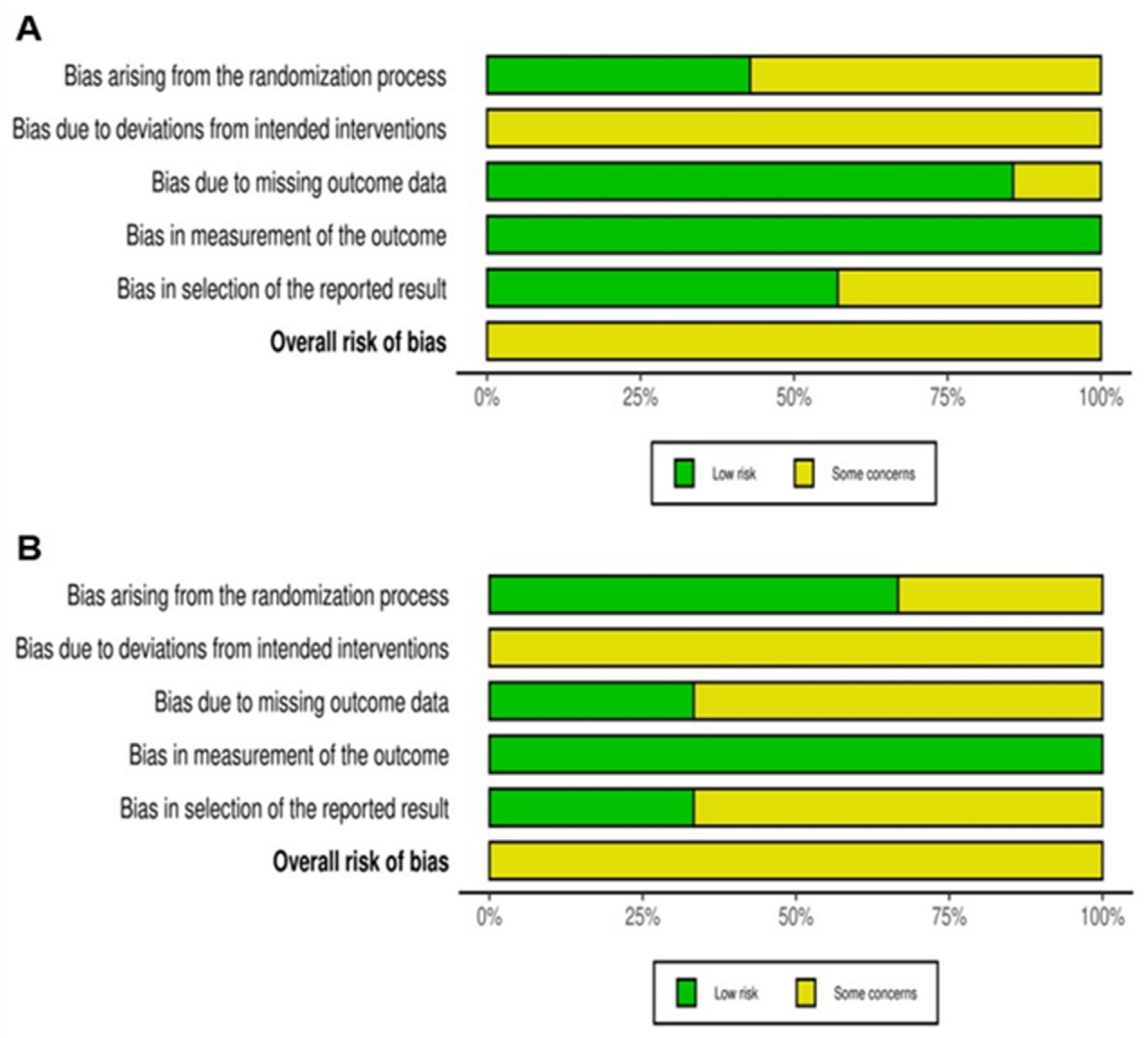
Summary risk of bias assessment of randomized controlled trials reporting the effect of isoenergetic replacement of cheese with other dairy products on blood lipid markers in the (A) fasted and (B) postprandial state using the Cochrane Risk of Bias tool 2.0.

The results from the overall RoB assessment are presented in Supplemental Figure 1. All 7 fasted state and 3 non-fasted state RCTs (100%) presented “some concerns” in ≥1 domain. With the exception of one RCT [46], all included crossover studies were judged to have an acceptable washout period between interventions (≥14 d). The RoB 2.0 domains that most contributed to “some concerns” were bias arising from the randomization process (D1) and bias because of deviations from intended interventions (D2) (Figure 2).

### Trials comparing cheese with butter intake on fasting blood lipid outcomes (meta-analysis)

**Figure 3:**
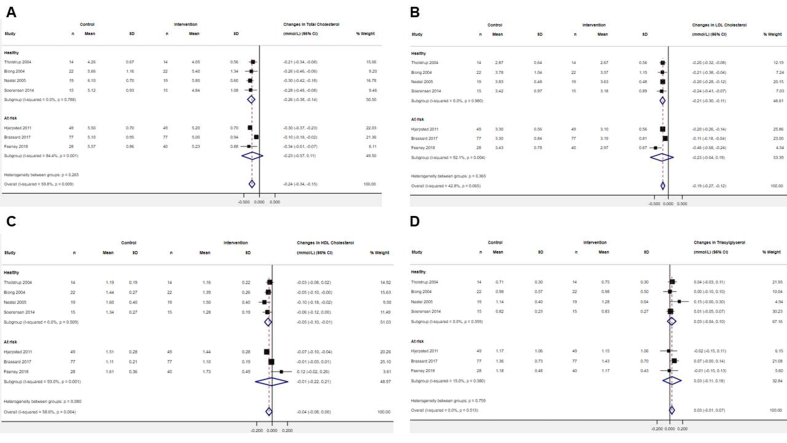
Forest plots of the effect of isoenergetic substitution of cheese with butter on fasting circulating (A) total cholesterol, (B) LDL-cholesterol, (C) HDL-cholesterol, (D) triacylglycerol in randomized controlled trials. Values were calculated as weighted mean differences (95% CIs) using an inverse variance random-effects model. The restricted maximum likelihood method was employed to estimate heterogeneity variance. The Hartung–Knapp–Sidik–Jonkman correction was applied to estimate the 95% CIs of the summary effects. CI, confidence interval.

Pooled analyses of 7 RCTs including 264 participants showed that ≥14 d mean daily intake of 135 g cheese reduced TC, LDL-C, and HDL-C concentrations (WMD: −0.24 mmol/L; 95% CI: −0.34, −0.15; *I*^2^ = 59.8%, WMD: −0.19 mmol/L; 95% CI: −0.27, −0.12; *I*^2^ = 42.8%, and WMD: −0.04 mmol/L; 95% CI: −0.08, −0.00; *I*^2^ = 58.6%, respectively), when compared with ∼52 g/d butter intake, even with evidence of moderate to substantial statistical heterogeneity (Figure 3). Relative to butter intake, subgroup analyses on TC and HDL-C concentrations revealed suggestive evidence of a lowering effect among the healthy subgroup after cheese intake (WMD: −0.26 mmol/L; 95% CI: −0.38, −0.14; *I*^2^ = 0.0% and WMD: −0.05 mmol/L; 95% CI: −0.10, −0.01; *I*^2^ = 0.0%, respectively; *n* = 4). Estimates in the “at risk” subgroup straddled the null, but the direction of effect was similar to the healthy subgroup (WMD: −0.23 mmol/L; 95% CI: −0.57, 0.11; *I*^2^ = 84.4% and WMD: −0.01 mmol/L; 95% CI: −0.22, 0.21; *I*^2^ = 93.0%, respectively; *n* = 3). Relative to butter intake, subgroup analysis on LDL-C concentrations provided suggestive evidence of a lowering effect among the “healthy” subgroup (WMD: −0.21 mmol/L; 95% CI: −0.30, −0.11; *I*^2^ = 0.0%; *n* = 4). There was a lowering effect in the “at risk” subgroup after cheese intake (WMD: −0.23 mmol/L; 95% CI: −0.64, 0.19; *I*^2^ = 92.1%; *n* = 3) but estimates straddled the null. No significant overall, or subgroup effect, of cheese compared with butter was observed on TG concentrations (WMD: 0.03 mmol/L; 95% CI: −0.01, 0.07; *I*^2^ = 0.0%, *n* = 7) (Figure 3). There was suggestive evidence of a lowering effect of cheese intake compared with butter on the LDL-C:HDL-C ratio (WMD: −0.08; 95% CI: −0.11, −0.05; *I*^2^ = 0.0%; *n* = 2) and apoB (WMD: −0.03 g/L; 95% CI: −0.07, −0.01; *I*^2^ = 0.0%; *n* = 3) (Supplemental Figure 2). There was no significant overall effect of cheese intake, when compared with butter, on apoA-I concentrations (WMD: −0.05 g/L; 95% CI: −0.30, 0.21; *I*^2^ = 0.0%; *n* = 2) or the TC:HDL-C ratio (WMD: −0.06; 95% CI: −0.56, −0.44; *I*^2^ = 0.0%; *n* = 2) (Supplemental Figure 2).

### Trials comparing cheese with butter intake on fasting blood lipid outcomes (narrative synthesis)

Of the studies included in meta-analyses, additional outcomes, specifically HDL 2 cholesterol (HDL_2_-C), HDL 3 cholesterol (HDL_3_-C), VLDL-C, lipoprotein (a), and nonesterified fatty acid (NEFA) concentrations, were each only reported in 1 RCT [[44], [45], [46]] and are therefore reported in narrative synthesis (Table 2, Supplemental Table 4). In apparently healthy Danish adults, Tholstrup et al. [45] found that plasma concentrations of HDL_2_-C, HDL_3_-C, and VLDL-C were similar after hard cheese (Samsø) and butter treatments. In a cohort of apparently healthy Norwegian adults, Biong et al. [46] found that lipoprotein (a) concentrations were similar after hard cheese (Jarlsberg) and butter interventions. Among overweight middle-to-older-aged Irish adults, Feeney et al. [44] found that NEFA concentrations were similar after hard cheese (Cheddar) and energy- and nutrient-matched intake of butter.

### Trials comparing cheese with milk intake on fasting blood lipid outcomes (meta-analysis)

The effect of the mean daily intake of 163 g cheese intake (163 g/d) compared with isoenergetic substitution with milk (1085 mL/d) on fasting blood lipid outcomes was investigated in 2 small crossover RCTs with only 29 participants. Pooled analyses, which require cautious interpretation, suggested that TC, LDL-C, HDL-C, and TG concentrations were similar after the dairy treatments (Supplemental Figure 3).

### Trials comparing cheese with milk intake on fasting blood lipid outcomes (narrative synthesis)

Additional outcomes, specifically HDL_2_-C, HDL_3_-C, and VLDL-C concentrations, were reported in one RCT by Tholstrup et al. [45]. The authors found no significant differences in these outcomes after isoenergetic quantities of hard cheese (Samsø) and butter (Table 2, Supplemental Table 4).

### Trials comparing cheese with other dairy products on postprandial blood lipid outcomes (narrative synthesis)

#### Cheese compared with butter

Of the included 3 postprandial studies, 2 RCTs compared isoenergetic replacement of cheese with butter on postprandial lipid outcomes [49,50] (Table 3). In apparently healthy Canadian adults, Drouin-Chartier et al. [50] found that Cheddar cheese and butter induced a similar increase in change-from-baseline in TG, NEFA, and apoB-48 concentrations over a 4-h postprandial period. Furthermore, no differences in the iAUC_0-8 h_ for TG, NEFA, and apoB-48 were observed between the meals (RoB: some concerns). The second study by Hansson et al. [49], compared fat-matched meals (45 g dairy fat) containing Gräddost (semi-hard cheese) or butter among Norwegian adults classified as healthy weight or overweight/obese. The authors found no significant cheese compared with butter meal effect on the postprandial TG, NEFA, TC, LDL-C, and iAUC_0-6 h_ (RoB: some concerns) [49].

#### Cheese compared with cream cheese or homogenized cheese

Two studies compared the replacement of Cheddar cheese with cream cheese (soft) or homogenized cheese (semi-solid) on postprandial lipid outcomes [15,50] (Table 3). Drouin-Chartier et al. [50] found no change in TG or NEFA percentage change in concentrations from baseline to 4 h or percentage difference in iAUC_0–8 h_. A significant increase in TG concentration was observed at 2 h after the cream cheese relative to the Cheddar cheese meal (change from baseline: +44% compared with +16%; *P* = 0.0004), whereas the TG response was attenuated at 6 h after cream cheese compared with the response induced by the Cheddar cheese meal (change from baseline: +14% compared with +42%; *P* = 0.0004). The NEFA response was lower at 2 h after the cream cheese relative to the Cheddar cheese meal (change from baseline: −57% compared with −68%; *P* = 0.04). The apoB-48 iAUC_0–8 h_ and apoB-48 concentrations at 4 and 6 h postprandially were lower after cream cheese, when compared with the Cheddar cheese meal (change from baseline at 4 h: +42% compared with +62% ; *P* = 0.02 and change from baseline at 6 h: +14% compared with +40% ; *P* = 0.0002, respectively) [50]. The second study by Kjølbæk et al. [15] found no difference in the iAUC_0-8 h_ or 8 h concentrations for TG, apoB-48, TC, HDL-C, LDL-C, NEFA, or apoB-100 after Cheddar cheese and homogenized Cheddar cheese meals.

#### Cheese compared with sour cream or whipped cream

Hansson et al. [49] also compared fat-matched meals containing Gräddost (semi-hard cheese) with sour cream and whipped cream (Table 3). A significant meal effect was evident in the iAUC_0-6 h_ for TG and HDL-C, with the intake of sour cream (but not whipped cream) inducing a +23% and +67% larger response relative to medium-hard cheese intake (*P* = 0.02 and *P* = 0.01, respectively) [49]. The authors [49] reported no significant difference in the iAUC_0-6 h_ for NEFA, TC or LDL-C after cheese, when compared with sour cream or whipped cream (RoB: some concerns).

## Discussion

This systematic review with meta-analysis assessed the effects of cheese intake compared with isoenergetic substitution with other dairy foods on blood lipid markers in the fasted and postprandial state. In our meta-analysis, we found that short-term (14–42 d) consumption of hard- or semi-hard cheese (mean daily intake: 135 g) lowered fasting circulating TC and LDL-C, and to a lesser extent HDL-C, relative to butter intake (∼52 g/d), even with evidence of statistical heterogeneity. There was suggestive evidence of a lowering effect of cheese intake compared with butter on the LDL-C:HDL-C ratio and apoB concentrations. There was no observed effect of this dairy fat substitution on other fasting lipid markers, including TG, ApoA-I concentrations, or the TC:HDL-C ratio. In line with a previous narrative synthesis (*n* = 2 RCTs) [13], we found no evidence of a benefit from 14 to 21 d replacement of cheese (mean daily intake: 163 g) with 1085 mL/d milk on fasting blood lipid markers, although findings were only based on pooled analyses of the same 2 RCTs. As outlined in narrative synthesis, evidence on the postprandial blood lipid effects of cheese relative to other dairy food structures is limited and insufficient to draw firm conclusions.

The present SRMA extends knowledge [13] by demonstrating that cheese consumption had a lesser effect in elevating TC, LDL-C, and HDL-C concentrations than an equivalent amount of butter in pooled analysis of 7 RCTs. Prospective epidemiological studies have shown that elevated circulating HDL-C is inversely associated with coronary heart disease risk [51,52]. However, more recently, the concept that elevated HDL-C concentrations will consistently translate into CVD risk reduction has been questioned [53], with Mendelian randomization analyses and large-scale clinical trial data indicating that HDL-C may not be casual in the etiology of coronary artery disease (CAD) [54,55]. Epidemiological evidence challenges the concept that raising HDL-C concentrations will uniformly translate into prognostic and therapeutic benefits, partly because circulating levels of this lipoprotein do not reflect HDL function [54,56]. HDL is understood to play an important role in the reverse cholesterol transport process by promoting the efflux of excess cholesterol from macrophages to the liver for biliary excretion and is inversely associated with risk of atherosclerotic CVD [57]. The secondary analysis [58] of the RCT by Brassard et al. [32], conducted among adults with abdominal obesity and relatively low baseline HDL-C concentrations, examined the impact of SFAs from cheese and butter on HDL-mediated cholesterol efflux capacity (CEC), as assessed ex vivo using radiolabeled J774 macrophages incubated with apoB-depleted sera (representing the pure HDL fraction). The authors reported that 4-wk consumption of a butter-rich diet increased ex vivo HDL-mediated CEC (an HDL functionality marker) to a greater extent than cheese and concluded that this finding may have been because of higher serum LDL-C, or possibly cholesterol derivatives (for example, oxysterols) [58]. These findings suggest that increased HDL-C concentrations (more specifically HDL-mediated CEC) could be a compensatory mechanism to offset the LDL-C raising effects induced by SFA in butter rather than as cheese, and lend support to the hypothesis that the dairy food matrix influences the relationship between SFA and CAD risk [58,59]. Accordingly, in the RCTs pooled in the current report, it could be speculated that there were underlying increases in HDL functional capacity to help offset the adverse effect of butter on circulating LDL-C. However, this would require confirmation in suitably designed human RCTs with biomarkers of HDL functionality, including HDL efflux capacity [53].

Our observation that dairy fat in the form of cheese attenuated the fasting cholesterol profile compared with an isocaloric quantity of butter could be attributed to a combination of factors, including the Ca, phosphorus, MFGM, and starter culture content of the cheese matrix (for detailed review, see [9,60]). As discussed previously by de Goede et al. [13], the relative cholesterol-lowering effect of cheese compared with butter could be, in part, explained by its Ca content, with one of our included RCTs [48] indicating that 2-wk intake of semi-hard cheese- (and milk-) rich diets (both 1700 mg Ca/d) led to increased fecal fat excretion and attenuated the SFA-induced increases in circulating TC and LDL-C compared with fat-matched butter intake (∼500 mg Ca/d). It has been shown that Ca, particularly dairy Ca, may increase fecal fat excretion by binding with free FAs in the intestine to form insoluble Ca soaps [61]. Indeed, a meta-analysis of 3 RCTs estimated that a short-term increase in dairy Ca intake by 1241 mg/d increased mean fecal fat excretion by 5.2 g/d relative to the low-Ca (<700 mg/d) dairy diet [61]. In the current report of 7 RCTs that compared cheese to butter intake, dairy or total Ca intake was not reported or matched across treatments in 6 of the included studies [31,32,[45], [46], [47], [48]], with cheese intake providing ≤1979 mg/d of additional dairy Ca relative to the butter treatment in the earliest RCT [45]. The most recent RCT by Feeney et al. [44] was well matched for total Ca intake. However, the authors acknowledged that they had employed a non-dairy Ca supplement (CaCO_3_) in the butter arm, rather than a dairy form of Ca (for example, calcium phosphate [CaP]) [44]. Lorenzen and Astrup [62] conducted a crossover RCT where participants were assigned 4 isoenergetic 10-d dairy treatments low Ca or high Ca in combination with low- or high-fat dairy foods. Findings suggested that dairy Ca may attenuate the TC and LDL-C raising effect of dairy fat, without reducing HDL-C concentrations, which may be in part because of the observed increase in fecal fat and bile acid excretion [62]. It should also be noted that the nutritional intake of phosphorus was not matched across dairy treatments in the abovementioned study (specifically for the low- and high-Ca diets) [62], or in the RCTs included in the current report. In contrast to butter, milk and milk products, including cheese, are high in both Ca and phosphorus (primarily in the form of phosphates and phosphate esters) [9,63]. Supplementation with CaP was previously shown to increase fecal bile acid excretion and reduce circulating LDL-C in humans [64]. Dietary Ca and phosphate precipitate in the small intestine to form Ca–phosphate complexes (insoluble amorphous CaP) and the resulting bile acid precipitation and disturbance of enterohepatic circulation may have contributed to the attenuated LDL-C response in the current report [64,65].

Another component of the dairy food matrix that could have contributed to the differential fasting circulating cholesterol profile between cheese and butter treatments is differences in MFGM content between these 2 dairy foods, although data were not reported in the 7 included RCTs. The MFGM, and its bioactive polar lipid (phospholipids and sphingolipids) and protein components, can be substantially reduced by commercial processes such as mechanical churning to produce butter, whereas it is present in a higher amount in the cheese matrix [10]. Human data suggest that supplementation with MFGM or milk polar lipids may have a beneficial role in the regulation of blood lipid profile, including TC and LDL-C concentrations, with data from rodent models indicating that supplementation may decrease intestinal lipid absorption and hepatic cholesterol accumulation and triglyceride accumulation (for detailed review, see [9,14]).

Compositionally, milk fat contains >400 different, including individual SFAs that vary in carbon chain length [66]. According to McCance and Widdowson’s Composition of Foods Integrated Dataset 2021 [67], butter contains more of the main dietary SFAs (including lauric (12:0), myristic (14:0), and palmitic (16:0) acids) than hard- and semi-hard cheese (per 100 g food). However, of the 7 included RCTs that compared cheese and butter intake, only 1 study provided information about the main dietary SFAs in study foods [31], with data indicating that the proportion of palmitic (16:0) acid was greater in butter, relative to cheese (29.2 compared with 27.1% by weight of total FAs). Two other RCTs [45,46] reported that there were no clear differences in the proportions of short-, medium-, and long-chain SFAs in the fully controlled experimental diets, but they did not report on the FA composition of the cheese and butter per se. In a systematic review and regression analysis on the effects of SFAs on serum lipids and lipoproteins, Mensink et al. [68] predicted that, compared with a mixture of carbohydrates, an increased intake of 12:0, 14:0, and 16:0 would raise serum TC, LDL-C, and HDL-C levels. It could be speculated that modest differences in the proportion of individual SFAs between the cheese and butter interventions could partly explain why these dairy foods had differential effects on the fasting lipid profile. However, as discussed earlier in this review, it is also increasingly recognized that the dairy matrix, or structure, in which these SFAs are contained may influence the blood lipid response [9].

There was an LDL-C lowering effect in the subgroup “at risk” of CVD after intake of dairy fat contained within the cheese matrix, compared with butter, although estimates straddled the null. This stratification included cohorts classified as overweight [44], those with elevated LDL-C concentrations [47], or abdominal obesity and low HDL-C concentrations [32] at baseline. Although our “at risk” stratification was broader, one of the RCTs included in this subgroup analysis [32] also indicated that the LDL-C-raising effects of dairy fats from butter relative to cheese is increased in individuals with higher baseline serum LDL-C. As discussed elsewhere, the inter-individual variation in LDL-C response to specific sources of SFAs requires further attention [6,69,70], as an increased understanding of the metabolic origins of this variation has the potential to advance the development of dietary guidelines that are tailored according to LDL-C responsiveness [71].

Exaggerated non-fasting concentrations of TG are an independent CVD risk factor, with high-fat meal intake known to lead to a transient increase in TG-rich lipoproteins during the postprandial period [21,72]. As the majority of the day is spent in the postprandial state [21,27], and the postprandial handling of TG may be a better CVD risk predictor than fasting TG concentrations [73], understanding of the postprandial lipid responses to meals varying in dairy matrix structure is of importance. Our narrative synthesis indicated that intake of cheese-rich meals may induce differential fed-state lipid responses compared with some other dairy matrix structures with similar nutritional content, including homogenized cream cheese and sour cream. However, the evidence based on 3 RCTs is very uncertain. Drouin-Chartier et al. [50] observed that consumption of a meal rich in homogenized cream cheese (a product that contains small, homogenized lipid droplets enclosed in a soft, semi-solid protein gel) induced a larger TG response during the early postprandial (first 2 h) period, without differences in AUC/iAUC_0–8 h_, and attenuated the iAUC_0–8 h_ for apoB-48 (a marker of intestinal chylomicron particles) relative to Cheddar cheese (a solid, intertwined matrix of fat globules and protein). Hansson et al. [49] reported that consumption of a meal rich in homogenized sour cream (a fermented dairy food) increased the iAUC_0-6 h_ for TG when compared with Gräddost (a semi-hard cheese). Homogenization of milk fat to produce processed cream, cheese, and sour cream leads to a substantial decrease in the size of fat droplets [49,74,75]. The physicochemical structures of fat in the homogenized cream cheese and sour cream meals may have led to enhanced fed-state TG responses (even only during the early postprandial period in Drouin-Chartier et al.), compared with hard or semi-hard cheese comparators [49,50], as lower initial size of fat droplets have a larger surface area for lipases to facilitate fat digestion and absorption [76,77]. Although the results of our narrative review suggest that the food source of dairy may modulate postprandial lipid response, further research is needed to confirm these findings.

This systematic literature review builds on the review of de Goede et al. 2015 [13] by presenting up-to-date evidence on the causal effects of cheese on lipid markers, relative to isoenergetic quantities of other dairy foods, in the fasted state. We used the REML method to estimate heterogeneity variance, with an estimated summary effect of the meta-analysis and its 95% CIs derived from the HKSJ method. These methods are recommended for meta-analyses with smaller sample sizes because they are considered more robust to changes in heterogeneity than traditional random-effect modeling according to DerSimonian and Laird [38], which was employed by de Goede et al. [13]. For the cheese compared with butter comparison, we pooled analyses from 264 participants across 7 RCTs for fasting TC, LDL-C, HDL-C, and TG outcomes, when compared with 100–119 participants across 4–5 RCTs in the former review by de Goede et al. [13]. We also performed subgroup analyses based on baseline CVD risk status, which is considered a novel aspect of our review. Furthermore, we extended on the former SRMA [13] assessing the effect of isoenergetic substitution of cheese with other dairy products on postprandial blood lipid biomarkers. Our updated and extended SRMA also benefits from adhering to recommendations from the Cochrane Handbook and PRISMA guidelines, including the RoB assessment. Nevertheless, some limitations also need to be acknowledged. First, we searched 3 databases and did not search grey literature, which is considered a limitation. Second, with the small number of included studies, our meta-analysis was limited to fasting studies that compared the effects of cheese compared with butter or milk on blood lipid outcomes. Because the meta-analyses comparing cheese with milk intake on fasting blood lipid outcomes only included 2 studies (pooled data from *n* = 29 participants), these results should be interpreted with caution. Third, we observed substantial heterogeneity in meta-analyses among some outcomes, explained by factors including differences in study methods/participants, which could have prevented the detection of statistically significant effect sizes. In line with Cochrane Handbook for Systematic Reviews recommendations [33], we used correlation coefficients to estimate intervention effects in crossover studies; however, this approach may have contributed to underestimated CIs of the effects from individual RCTs [29]. It was not feasible to carry out a meta-analysis on specific postprandial lipid outcomes because of methodological heterogeneity across the limited included studies, with the main issue related to the way results were reported. Our meta-analyses revealed that cheese intake (mean daily intake: 135 g) achieved a mean reduction of 0.19 mmol/L in circulating LDL-C, relative to butter intake (∼52 g/d). This reduction is of public health importance given that an LDL-C reduction of 1.0 mmol/L has been associated with a 19% lower risk of coronary mortality in a prospective meta-analysis [78]. However, it should be acknowledged that the majority of included studies involved the provision of large quantities of dairy foods, which may not reflect habitual dairy and total fat intake [13,79]; this point should be taken into consideration when interpreting and drawing conclusions from the current review.

## Recommendations for future research

Further research is needed to compare the lipid/lipoprotein effects of energy- and macronutrient-matched intake of regular fat cheese compared with fat-reduced cheese as this will help to inform future food-based dietary guidance. As discussed previously [80], it may be necessary to consider cheese as a diverse category of dairy foods that vary in composition and structure, according to type, milk pretreatment methods, and manufacturing and maturation processes. Although it is recognized that these factors have the potential to work collectively to influence digestive, nutritional, and subsequent health properties of a cheese, further research is needed to better understand the influence of the specific matrix on aspects of cardiometabolic health [80]. To provide a more comprehensive overview of the impact of the dairy food matrix structure on CVD risk, more research is needed on the food composition of dairy treatments with respect to micronutrient (including Ca and phosphorus), individual SFAs, and MFGM content. Future work should also consider emerging lipid-related biomarkers, including LDL and HDL particle size distribution and subclass phenotype [81]. For example, it is becoming increasingly recognized that a higher prevalence of small dense LDL particles is more predictive of CVD risk when compared with LDL-C concentrations [82]. Finally, as high-fat meals induce prolonged TG elevation, it is recommended that future studies examining dairy matrix structure should capture postprandial lipid responses over an 8–10-h period [26,27]. In line with the study design of Kjølbæk et al. [15], RCTs should match for fat and protein, as the characteristics of the latter macronutrient are also an important determinant of postprandial metabolic effects [83].

## Conclusion

In conclusion, this meta-analysis provides evidence that, relative to butter, intake of hard or semi-hard cheese for ≥14 d lowered fasting circulating TC and LDL-C, and to a lesser extent HDL-C among adults. Our findings suggest that the cheese matrix may modulate the effect of dairy fat on the circulating lipoprotein profile. However, owing to considerable heterogeneity and limited studies, further confirmation from RCTs is warranted. More research is needed to determine whether specific aspects of the cheese matrix can provide an explanation for this observation.

## Author contributions

The authors’ responsibilities were as follows—RP, OM: developed the systematic review protocol and database search strategy; RP: performed the literature searches; RP, OM: contributed to the independent screening of titles, abstracts and full-text records, extracted data, and performed risk of bias assessment and synthesis; RP, TN, OM: performed data harmonization; RP, TN, LS, OM: contributed to the data analysis plan; TN: conducted the meta-analyses; OM: wrote the first draft of the manuscript; RP, TN, LS: provided critical review of important intellectual content and contributed to the interpretation of the data; OM: had primary responsibility for the final content; and all authors: reviewed and commented on versions of the manuscript and read and approved the final manuscript.

## Conflict of interest

OM has received research funding from Arla Food Ingredients, Denmark. All other authors report no conflicts of interest.

## Funding

There was no funding received from any organization to complete this research.
