# Supplementary material for: Effect of Isoenergetic Substitution of Cheese with Other Dairy Products on Blood Lipid Markers in the Fasted and Postprandial State: An Updated and Extended Systematic Review and Meta-Analysis of Randomized Controlled Trials in Adults
Source: Adv Nutr. 2023 Sep 17;14(6):1579–95. doi: 10.1016/j.advnut.2023.09.003 (PMC10721513; doi:10.1016/j.advnut.2023.09.003)
Supplement: Multimedia component 1 [file mmc1.docx]

**Effect of Isoenergetic Substitution of Cheese with Other Dairy Products on Blood Lipid Markers in the Fasted and Postprandial State: An Updated and Extended Systematic Review and Meta-analysis of Randomized Controlled Trials in Adults**

Pradeilles et al.

Online Supplementary Material

**Supplemental Table 1** Database searches

**Database:** PubMed (Medline)

**Date:** 15 June 2022

**Results** 1,008

("cheese"[MeSH Terms] OR "cheese"[All Fields] OR "cheeses"[All Fields] OR "cheese s"[All Fields] OR ("cheese"[MeSH Terms] OR "cheese"[All Fields] OR "cheeses"[All Fields] OR "cheese s"[All Fields])) AND ("triglyceride*"[All Fields] OR "tg"[All Fields] OR "chol"[All Fields] OR ("cholesterol"[MeSH Terms] OR "cholesterol"[All Fields] OR "cholesterol s"[All Fields] OR "cholesterole"[All Fields] OR "cholesterols"[All Fields]) OR "lipoprotein*"[All Fields] OR "apolipoprotein*"[All Fields] OR ("lipoprotein a"[MeSH Terms] OR "lipoprotein a"[All Fields] OR "lp a"[All Fields]) OR "lpa"[All Fields] OR ("lipoprotein a"[MeSH Terms] OR "lipoprotein a"[All Fields] OR "lipoprotein a"[All Fields]) OR ("apolipoproteins b"[MeSH Terms] OR "apolipoproteins b"[All Fields] OR "apob"[All Fields]) OR ("apolipoproteins a"[MeSH Terms] OR "apolipoproteins a"[All Fields] OR "apoa"[All Fields]) OR "vldl*"[All Fields] OR "ldl"[All Fields] OR "hdl"[All Fields] OR ("cholesterol"[MeSH Terms] OR "cholesterol"[All Fields] OR "cholesterol s"[All Fields] OR "cholesterole"[All Fields] OR "cholesterols"[All Fields]) OR "lipid*"[All Fields] OR "hypertriglycerid*"[All Fields] OR "hyperlipidaemia*"[All Fields] OR "hyperlipidemia*"[All Fields] OR "dyslipidaemia*"[All Fields] OR "dyslipidemia*"[All Fields] OR "hypercholester*"[All Fields] OR "triacylglycerol*"[All Fields])

**Database:** Cochrane Central

**Date:** 16 June 2022

ID Search Hits

#1 (cheese):ti,ab,kw (Word variations have been searched) 772

#2 (triglyceride*):ti,ab,kw (Word variations have been searched) 23963

#3 (TG*):ti,ab,kw (Word variations have been searched) 11055

#4 (chol):ti,ab,kw (Word variations have been searched) 362

#5 (Cholesterol):ti,ab,kw (Word variations have been searched) 39188

#6 (Lipoprotein*):ti,ab,kw (Word variations have been searched) 24751

#7 (apolipoprotein*):ti,ab,kw (Word variations have been searched) 5309

#8 (lp):ti,ab,kw (Word variations have been searched) 2991

#9 (lpa):ti,ab,kw (Word variations have been searched) 1164

#10 (lipoprotein a):ti,ab,kw (Word variations have been searched) 22906

#11 (apob):ti,ab,kw (Word variations have been searched) 1302

#12 (apoa):ti,ab,kw (Word variations have been searched) 547

#13 (VLDL*):ti,ab,kw (Word variations have been searched) 2235

#14 (LDL*):ti,ab,kw (Word variations have been searched) 22956

#15 (HDL*):ti,ab,kw (Word variations have been searched) 18351

#16 (cholesterol):ti,ab,kw (Word variations have been searched) 39188

#17 (lipid*):ti,ab,kw (Word variations have been searched) 47445

#18 (hypertriglycerid*):ti,ab,kw (Word variations have been searched) 2395

#19 (hyperlipidaemia*):ti,ab,kw (Word variations have been searched) 642

#20 (hyperlipidemia*):ti,ab,kw (Word variations have been searched) 5995

#21 (dyslipidaemia*):ti,ab,kw (Word variations have been searched) 754

#22 (dyslipidemia*):ti,ab,kw (Word variations have been searched) 5980

#23 (hypercholester*):ti,ab,kw (Word variations have been searched) 8473

#24 (triacylglycerol*):ti,ab,kw (Word variations have been searched) 8697

#25 MeSH descriptor: [Cheese] this term only 96

#26 MeSH descriptor: [Triglycerides] this term only 6605

#27 MeSH descriptor: [Cholesterol] this term only 6112

#28 MeSH descriptor: [Lipoproteins] this term only 2092

#29 MeSH descriptor: [Apolipoproteins] this term only 565

#30 MeSH descriptor: [Cholesterol, LDL] this term only 4959

#31 MeSH descriptor: [Cholesterol, VLDL] this term only 257

#32 MeSH descriptor: [Cholesterol, HDL] this term only 3851

#33 MeSH descriptor: [Hyperlipidemias] this term only 2046

#34 MeSH descriptor: [Dyslipidemias] this term only 1364

#35 MeSH descriptor: [Hypertriglyceridemia] this term only 694

#36 MeSH descriptor: [Hypercholesterolemia] this term only 3590

#37 #2 OR #3 OR #4 OR #5 OR #6 OR #7 OR #8 OR #9 OR #10 OR #11 OR #12 OR #13 OR #14 OR #15 OR #16 OR #17 OR #18 OR #19 OR #20 OR #21 OR #22 OR #23 OR #24 OR #26 OR #27 OR #28 OR #29 OR #30 OR #31 OR #32 OR #33 OR #34 OR #35 OR #36 89218

#38 #1 OR #25 772

#39 #37 AND #38 236

**Database:** Embase

**Date:** 15 June 2022

**Results** 1,038

('cheese'/exp OR cheese OR cheeses) AND (triglyceride*:ab,ti OR tg*:ab,ti OR chol:ab,ti OR lipoprotein*:ab,ti OR apolipoprotein*:ab,ti OR 'lp a':ab,ti OR lpa:ab,ti OR 'lipoprotein a':ab,ti OR apob:ab,ti OR apoa:ab,ti OR vldl*:ab,ti OR ldl*:ab,ti OR hdl*:ab,ti OR cholesterol:ab,ti OR lipid*:ab,ti OR hypertriglycerid*:ab,ti OR hyperlipidaemia*:ab,ti OR hyperlipidemia*:ab,ti OR dyslipidaemia*:ab,ti OR dyslipidemia*:ab,ti OR hypercholester*:ab,ti OR triacylglycerol*:ab,ti) AND english:la

**Supplemental Table 2** Data extraction form template

| **Study details** | | | | | | | | | | | | | |
| --- | --- | --- | --- | --- | --- | --- | --- | --- | --- | --- | --- | --- | --- |
| Initials of extractor | First author, year (Country) | | | | | Title of paper | | | |  | | | |
| **Participant characteristics and health status** | | | | | | | | | | | | | |
| Health status | | Age (mean or range) | | BMI (mean or range) | | | Sex (M or M+F) | | | | No. of participants who completed the study (% M/F) | | Randomization process |
| **Study design: Intervention and comparator** | | | | | | | | | | | | | |
| Study design | | No. of study arms | | | Type of intervention (Controlled/semi-controlled) | | | | Duration of intervention (d) | | | Duration of washout (d) (Requirements) | |
| Intervention (Description and quantity consumed) | | Type of intervention cheese | | | Comparator (Description and quantity consumed) | | | | Intake unit | | | Intake unit - other specify | |
| Duration of fast | | Controlled factors between treatments (relevant study arms only) | | | Method of dietary assessment | | | | Relevant outcomes | | | Methods of assessment (all relevant outcomes) | |
| Test meal (Description, including total energy and fat content)^1^ | | Total fat content of the test meal^1^ | | | Other test meal components^1^ | | | | Dietary intake standardized between meals (Yes/No)^1^ | | | Duration of study visit^1^ | |
| Blood sampling timepoints^1^ | | Primary outcome | | | Power calculation details | | | | Text summarizing statistical approach | | | Body mass similar between relevant study arms post-intervention (Yes/No) | |
| **Outcome measures and results** | | | | | | | | | | | | | |
| Comparison | | Outcome (units) | | | Analytical sample (*n*) | | | |  | | |  | |
| Mean values at baseline (± SD); or mean % prevalence at baseline (95% CIs) | | Mean values at endline/follow-up (± SD); or mean % prevalence post-intervention (95% CIs) | | | Change in mean value baseline to follow-up/endline (± SE/SD) or change in % prevalence from baseline to post-intervention (95% CI) | | | | P-value | | |  | |
| Adjusted values? (Yes/No) – If Yes, specify | | Confounders (list all) | | | Text summarizing all relevant outcomes | | | |  | | |  | |
| **Additional information** | | | | | | | | | | | | | |
| Authors' conclusion | | | Strengths and limitations of study | | | | | Other notes (include relevant references) | | | | | |
| Notes (e.g., highlight missing information/issues with extraction etc. | | | Industrial funding source (Yes/No/Not stated) | | | | | Authors’ declaration of interests | | | | | |

^1^Information extracted from postprandial studies only.

**Supplemental Table 3** Approach to reaching overall risk of bias judgement for randomized controlled trials using the Cochrane Risk of Bias tool 2.0^1^

| **Overall risk of bias judgement** | **Criteria** |
| --- | --- |
| Low risk of bias | The study is judged to be at low risk of bias for all domains for this result |
| Moderate risk of bias (Some concerns) | The study is judged to raise some concerns in at least one domain for this result, but not to be at high risk of bias for any domain |
| High risk of bias | The study is judged to be at risk of bias in at least one domain for this result, or the study is judged to have some concerns for multiple domains in a way that substantially lowers confidence in the result |

^1^From Sterne et al 2019 (1).

**Supplemental Table 4** Synthesis of results of eligible RCTs on the impact of isoenergetic replacement of cheese with other dairy products on blood lipid markers in the fasted state^1^

| **First author, year (Country)** | **Study completers, *n*** | **Baseline values for cheese vs. comparator**  **(Mean ± SD, unless specified)** | **Post-intervention values for cheese vs. comparator**  **(Mean ± SD, unless specified)** |
| --- | --- | --- | --- |
| *Cheese vs. butter* | | | |
| Tholstrup, 2004 (Denmark) (2) | 14 | TC: 4.11 ± 0.16 mmol/L^1,2^  LDL-C: 2.64 ± 0.16 mmol/L^1,2^  HDL-C: 1.23 ± 0.06 mmol/L^1,2^  HDL_2_-C: 0.37 ± 0.03 mmol/L^1,2^  HDL_3_-C: 0.86 ± 0.04 mmol/L^1,2^  LDL-C:HDL-C ratio: 2.26 ± 0.20^1,2^  VLDL-C: 0.21 ± 0.03 mmol/L^1,2^  TG: 0.89 ± 0.09 mmol/L^1,2^  apoB: 0.86 ± 0.05 g/L^1,2^  apoA-I: 1.39 ± 0.04 g/L^1,2^ | TC: 4.05 ± 0.15 vs. 4.26 ± 0.18 mmol/L^2^  LDL-C: 2.67 ± 0.15 vs. 2.87 ± 0.17 mmol/L^2^  HDL-C: 1.16 ± 0.06 vs.1.19 ± 0.05 mmol/L^2^  HDL_2_-C: 0.32 ± 0.02 vs. 0.34 ± 0.03 mmol/L^1,2^  HDL_3_-C: 0.85 ± 0.04 vs. 0.85 ± 0.03 mmol/L^1,2^  LDL-C:HDL-C ratio: 2.41 ± 0.20 vs. 2.50 ± 0.19^2^  VLDL-C: 0.19 ± 0.03 vs. 0.17 ± 0.03 mmol/L^2^  TG: 0.75 ± 0.08 vs. 0.71 ± 0.08 mmol/L^2^  apoB: 0.87 ± 0.06 vs. 0.89 ± 0.05 g/L^2^  apoA-I: 1.27 ± 0.05 vs. 1.31 ± 0.04 g/L^2^ |
| Biong, 2004 (Norway) (3) | 22 | TC: 5.55 ± 1.17 mmol/L^1^  LDL-C: 3.61 ± 0.99 mmol/L^1^  HDL-C: 1.47 ± 0.29 mmol/L^1^  LDL-C:HDL-C ratio: 2.53 ± 0.79^1^  TG: 1.07 ± 0.65 mmol/L^1^  apoB: 0.93 ± 0.23 g/L^1^  apoA-I: 1.56 ± 0.28 g/L^1^  Lp(a) –^2^ | TC: 5.40 ± 1.34 vs. 5.66 ± 1.16 mmol/L  LDL-C: 3.57 ± 1.15 vs. 3.78 ± 1.04 mmol/L  HDL-C: 1.39 ± 0.26 vs. 1.44 ± 0.27 mmol/L  LDL-C:HDL-C ratio: 2.63 ± 0.87 vs. 2.71 ± 0.88  TG: 0.98 ± 0.50 vs. 0.98 ± 0.57 mmol/L  apoB: 0.92 ± 0.27 vs. 0.96 ± 0.25 g/L  apoA-I: 1.40 ± 0.21 vs. 1.45 ± 0.20 g/L  Lp(a): 132 ± 155 vs. 143 ± 154 mg/L |
| Nestel, 2005 (Australia) (4) | 19 | TC: 5.6 ± 0.8 mmol/L^1^  LDL-C: 3.4 (3.0–4.1) mmol/L^1,3^  HDL-C: 1.5 ± 0.4 mmol/L^1^  TG: 1.1 (0.7–1.4) mmol/L^1^ | TC: 5.8 ± 0.6 vs. 6.1 ± 0.7 mmol/L  LDL-C: 3.7 (3.3-3.9) vs. 3.9 (3.5-4.1) mmol/L^3^  HDL-C: 1.5 ± 0.4 vs. 1.6 ± 0.4 mmol/L  TG: 1.5 (0.8–1.6) vs. 1.1 (0.9–1.4) mmol/L^3^ |
| Hjerpsted, 2011 (Denmark) (5) | 49 | TC: 5.24 ± 0.70 mmol/L^1,2,5^  LDL-C: 3.17 ± 0.56 mmol/L^1,2,5^  HDL-C: 1.48 ± 0.28 mmol/L^1,2,5^  TC:HDL-C ratio: 3.57 ± 1.03^1,2,5^  TG: 1.06 ± 1.06 mmol/L^1,2,5^ | TC: 5.20 ± 0.70 vs. 5.50 ± 0.70^2,5^  LDL-C: 3.10 ± 0.56 vs. 3.30 ± 0.56^2,5^  HDL-C: 1.44 ± 0.28 vs. 1.51 ± 0.28^2,5^  TC:HDL-C ratio: 3.59 ± 1.03 vs. 3.68 ± 1.03^2^  TG: 1.15 ± 1.06 vs. 1.17 ± 1.06 mmol/L^2^ |
| Soerensen, 2014 (Denmark) (6) | 15 | TC: 4.43 ± 1.12 vs. 4.23 ± 0.81 mmol/L  LDL-C: 2.71 ± 0.74 vs. 2.57 ± 0.62 mmol/L  HDL-C: 1.33 ± 0.19 vs. 1.30 ± 0.27 mmol/L  TG: 0.85 ± 0.31 vs. 0.78 ± 0.23 mmol/L | TC: 4.84 ± 1.08 vs. 5.12 ± 0.93 mmol/L  LDL-C: 3.18 ± 0.89 vs. 3.42 ± 0.97 mmol/L  HDL-C: 1.28 ± 0.19 vs. 1.34 ± 0.27 mmol/L  TG: 0.83 ± 0.27 vs. 0.82 ± 0.23 mmol/L |
| Brassard, 2017 (Canada) (7) | 92 (47/53) - completed ≥1 diet (*n* = 77 post-intervention) | TC: 4.97 ± 0.97 vs. 4.96 ± 0.88 mmol/L  LDL-C: 3.09 ± 0.83 vs. 3.03 ± 0.76 mmol/L  HDL-C: 1.17 ± 0.25 vs. 1.18 ± 0.25 mmol/L  TC: HDL-C ratio: 4.41 ± 1.08 vs. 4.38 ± 1.20  TG: 1.41 ± 0.76 vs. 1.48 ± 0.76 mmol/L  ApoB: 1.67 ± 0.51 vs. 1.63 ± 0.47 g/L | TC: 5.00 ± 0.94 vs. 5.10 ± 0.95 mmol/L  LDL-C: 3.19 ± 0.81 vs. 3.30 ± 0.84 mmol/L  HDL-C: 1.10 ± 0.19 vs. 1.11 ± 0.21 mmol/L  TC: HDL-C ratio: 4.67 ± 1.04 vs. 4.73 ± 1.18  TG: 1.43 ± 0.70 vs. 1.36 ± 0.73 mmol/L  ApoB: 1.72 ± 0.50 vs. 1.74 ± 0.58 g/L |
| Feeney, 2018 (Ireland) (8) | Per-protocol analysis: 127 *n* = 40 and *n* = 28 for cheese and butter treatments, respectively | TC: 5.75 ± 1.09 vs. 5.72 ± 0.70 mmol/L  LDL-C: 3.42 ± 0.87 vs. 3.57 ± 0.66 mmol/L  HDL-C: 1.73 ± 0.49 vs. 1.56 ± 0.33 mmol/L  TG: 1.32 ± 0.57 vs. 1.30 ± 0.61 mmol/L  NEFA: 0.63 ± 0.30 vs. 0.62 ± 0.29 mmol/L | TC: 5.23 ± 0.88 vs. 5.57 ± 0.86 mmol/L  LDL-C: 2.97 ± 0.67 vs. 3.43 ± 0.78 mmol/L  HDL-C: 1.73 ± 0.49 vs. 1.61 ± 0.36 mmol/L  TG: 1.17 ± 0.43 vs. 1.18 ± 0.48 mmol/L  NEFA: 0.56 ± 0.23 vs. 0.61 ± 0.34 mmol/L |
| *Cheese vs. milk* | | | |
| Tholstrup, 2004 (Denmark) (2)^1^ | 14 | TC: 4.11 ± 0.16 mmol/L^1,2^  LDL-C: 2.64 ± 0.16 mmol/L^1,2^  HDL-C: 1.23 ± 0.06 mmol/L^1,2^  HDL_2_-C: 0.37 ± 0.03 mmol/L^1,2^  HDL_3_-C: 0.86 ± 0.04 mmol/L^1,2^  LDL:HDL-C ratio: 2.26 ± 0.20^1,2^  VLDL-C: 0.21 ± 0.03 mmol/L^1,2^  TG: 0.89 ± 0.09 mmol/L^1,2^  apoB: 0.86 ± 0.05 g/L^1,2^  apoA-I: 1.39 ± 0.04 g/L^1,2^ | TC: 4.05 ± 0.15 vs. 4.18 ± 0.17 mmol/L^1,2^  LDL-C: 2.67 ± 0.15 vs. 2.81 ± 0.18 mmol/L^1,2^  HDL-C: 1.16 ± 0.06 vs. 1.15 ± 0.07 mmol/L^1,2^  HDL_2_-C: 0.32 ± 0.02 vs. 0.34 ± 0.03 mmol/L^1,2^  HDL_3_-C: 0.85 ± 0.04 vs. 0.81 ± 0.05 mmol/L^1,2^  LDL:HDL-C ratio: 2.41 ± 0.20 vs. 2.62 ± 0.26  VLDL-C: 0.19 ± 0.03 vs. 0.19 ± 0.03 mmol/L^1,2^  TG: 0.75 ± 0.08 vs. 0.73 ± 0.08 mmol/L^1,2^  apoB: 0.87 ± 0.06 vs. 0.89 ± 0.05 g/L^1,2^  apoA-I 1.27 ± 0.05 vs. 1.31 ± 0.04 g/L^1,2^ |
| Soerensen, 2014 (Denmark) (6) | 15 | TC: 4.43 ± 1.12 vs. 4.26 ± 0.93 mmol/L  LDL-C: 2.71 ± 0.74 vs. 2.57 ± 0.62 mmol/L  HDL-C: 1.33 ± 0.19 vs. 1.35 ± 0.23 mmol/L  TG: 0.85 ± 0.31 vs. 0.76 ± 0.31 mmol/L | TC: 4.84 ± 1.08 vs. 4.83 ± 1.12 mmol/L  LDL-C: 3.18 ± 0.89 vs. 3.42 ± 0.97 mmol/L  HDL-C: 1.28 ± 0.19 vs. 1.33 ± 0.19 mmol/L  TG: 0.83 ± 0.27 vs. 0.91 ± 0.31 mmol/L |

^1^Only one set of mean fasting baseline values were reported.

^2^Values reported as SEM.

^3^Baseline value for Lp(a) was not analysed.

^4^Values reported as median (interquartile range).

^5^Values estimated from figure.

apo, apolipoprotein; HDL-C, HDL cholesterol; HDL_2_-C, HDL 2 cholesterol; HDL_3_-C, HDL 3 cholesterol; LDL-C, LDL cholesterol; Lp(a), lipoprotein (a); NEFA, nonesterified fatty acid; NR, not reported; TC, total cholesterol; TG, triacylglycerol; VLDL-C, VLDL cholesterol.


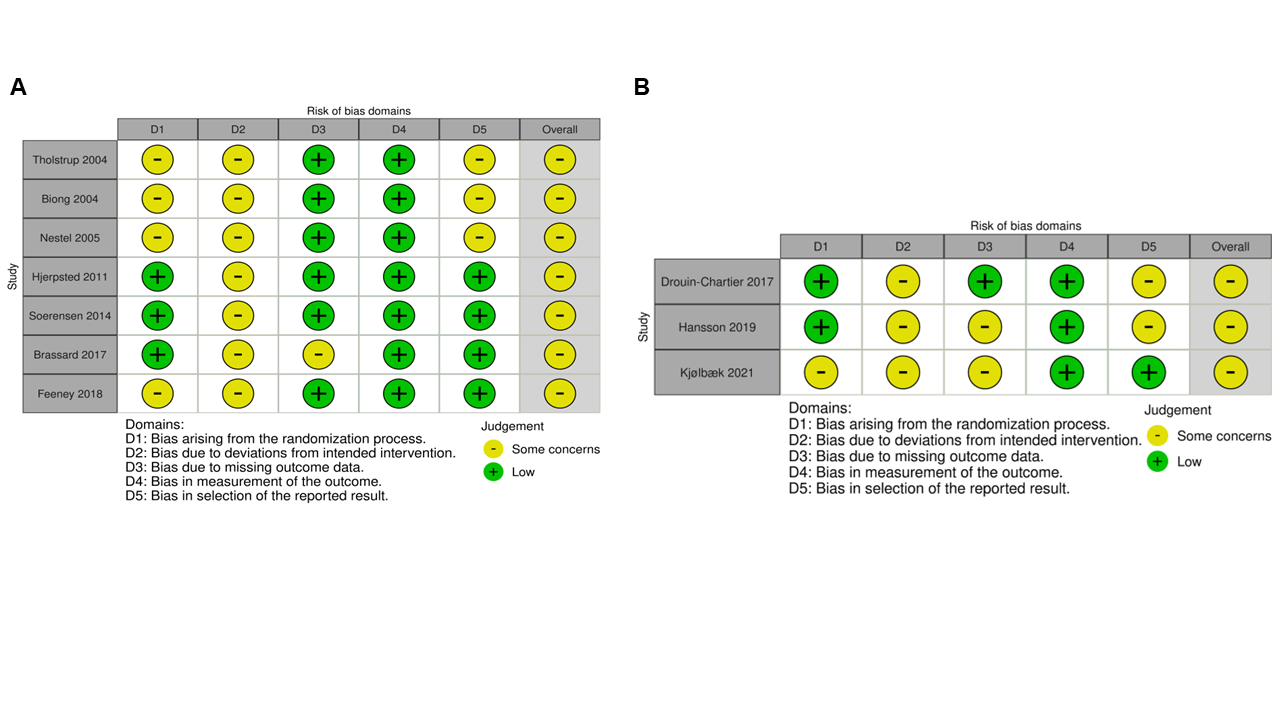


**Supplemental Figure 1** Risk of bias assessment of randomized controlled trials reporting the impact of isoenergetic substitution of cheese with other dairy products on blood lipid markers in the (a) fasted and (b) postprandial state using the Cochrane Risk of Bias tool 2.0 (1).


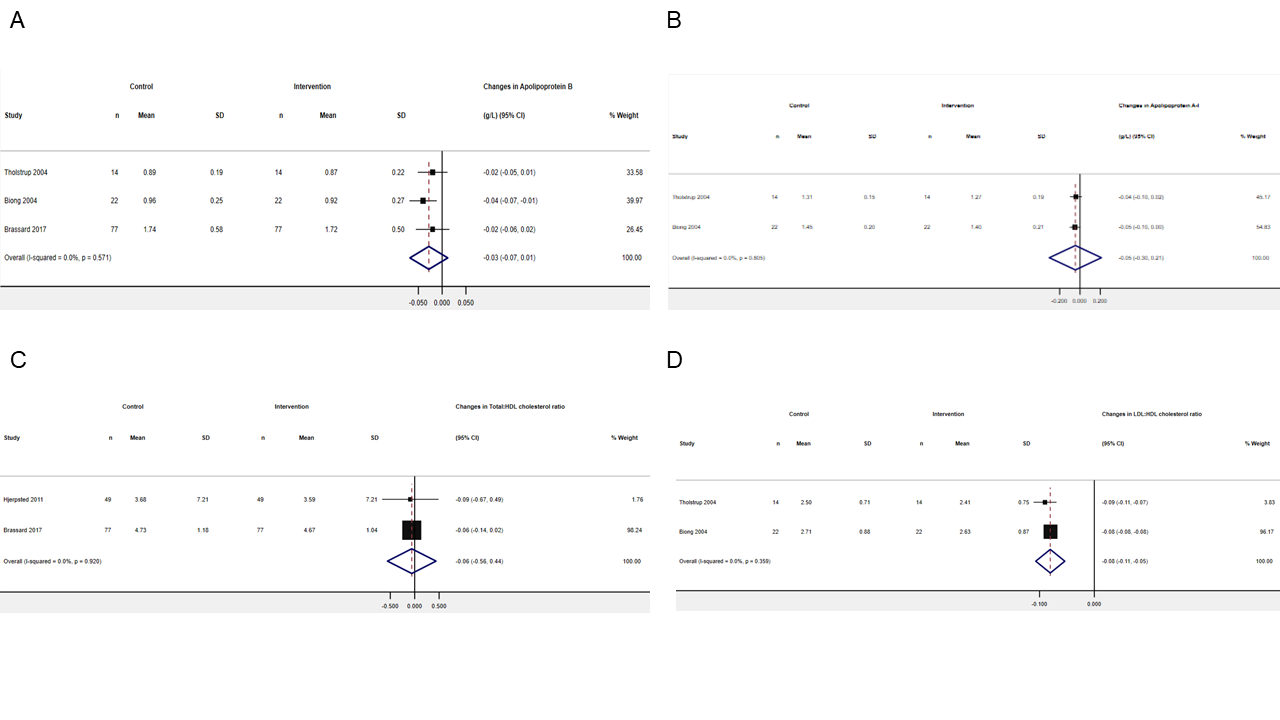
**Supplemental Figure 2** Forest plots of the effect of isoenergetic substitution of cheese with butter on fasting circulating (A) apolipoprotein B, (B) apolipoprotein A-I, (C) Total cholesterol:HDL-C ratio, and (D) LDL-cholesterol:HDL-cholesterol ratio in randomized controlled trials. Values were calculated as weighted mean differences (95% CIs) using an inverse variance random-effects model. The restricted maximum likelihood method was employed to estimate heterogeneity variance. The Hartung-Knapp-Sidik-Jonkman correction was applied to estimate the 95% CIs of the summary effects.


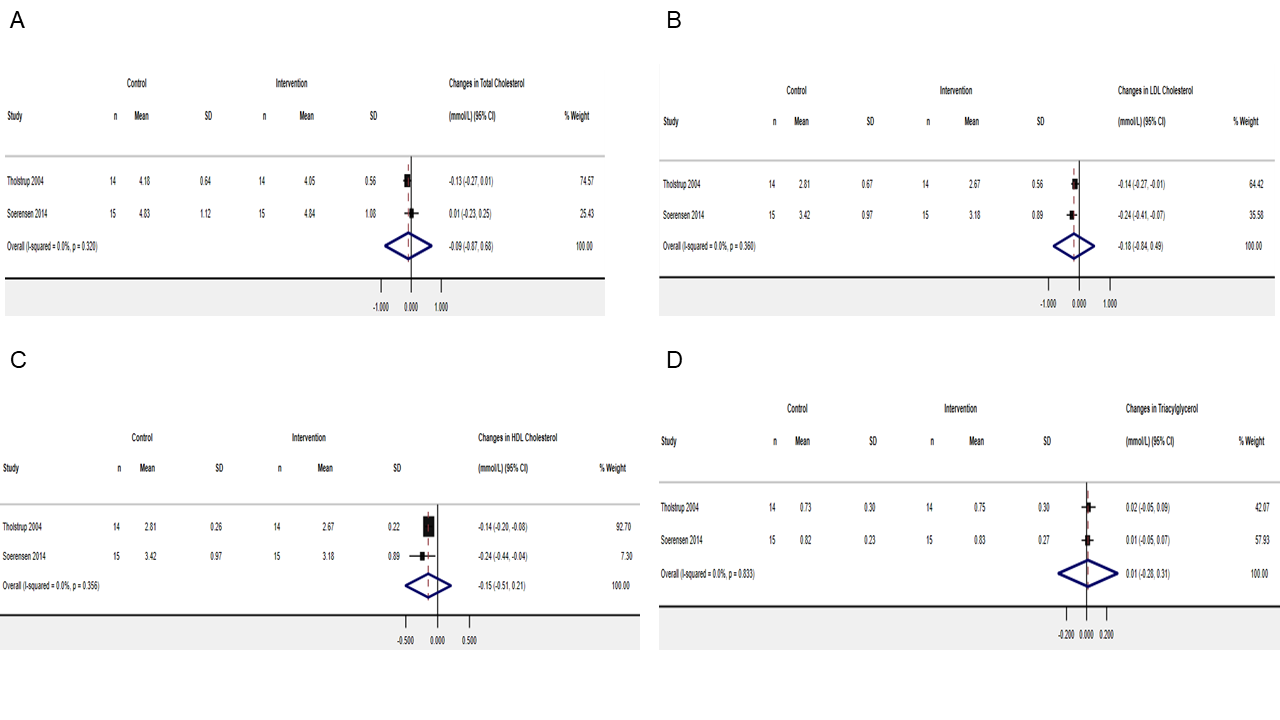


**Supplemental Figure 3** Forest plots of the effect of isoenergetic substitution of cheese with milk on fasting circulating (A) total cholesterol, (B) LDL-cholesterol, (C) HDL-cholesterol, (D) triacylglycerol in randomized controlled trials. Values were calculated as weighted mean differences (95% CIs) using an inverse variance random-effects model. The restricted maximum likelihood method was employed to estimate heterogeneity variance. The Hartung-Knapp-Sidik-Jonkman correction was applied to estimate the 95% CIs of the summary effects.

**Supplemental References**

1. Sterne JAC, Savović J, Page MJ, Elbers RG, Blencowe NS, Boutron I et al. RoB 2: a revised tool for assessing risk of bias in randomised trials. BMJ. 2019;366:l4898.

2. Tholstrup T, Høy C-E, Andersen LN, Christensen RD, Sandström B. Does fat in milk, butter and cheese affect blood lipids and cholesterol differently? J Am Coll Nutr. 2004;23(2):169-76.

3. Biong AS, Müller H, Seljeflot I, Veierød MB, Pedersen JI. A comparison of the effects of cheese and butter on serum lipids, haemostatic variables and homocysteine. Br J Nutr. 2004;92(5):791-7.

4. Nestel P, Chronopulos A, Cehun M. Dairy fat in cheese raises LDL cholesterol less than that in butter in mildly hypercholesterolaemic subjects. Eur J Clin Nutr. 2005;59(9):1059-63.

5. Hjerpsted J, Leedo E, Tholstrup T. Cheese intake in large amounts lowers LDL-cholesterol concentrations compared with butter intake of equal fat content. Am J Clin Nutr. 2011;94(6):1479-84.

6. Soerensen KV, Thorning TK, Astrup A, Kristensen M, Lorenzen JK. Effect of dairy calcium from cheese and milk on fecal fat excretion, blood lipids, and appetite in young men. Am J Clin Nutr. 2014;99(5):984-91.

7. Brassard D, Tessier-Grenier M, Allaire J, Rajendiran E, She Y, Ramprasath V et al. Comparison of the impact of SFAs from cheese and butter on cardiometabolic risk factors: a randomized controlled trial. Am J Clin Nutr. 2017;105(4):800-9.

8. Feeney EL, Barron R, Dible V, Hamilton Z, Power Y, Tanner L et al. Dairy matrix effects: response to consumption of dairy fat differs when eaten within the cheese matrix—a randomized controlled trial. Am J Clin Nutr. 2018;108(4):667-74.
